# Skin-interfaced microfluidic systems with spatially engineered 3D fluidics for sweat capture and analysis

**DOI:** 10.1126/sciadv.adg4272

**Published:** 2023-05-03

**Authors:** Chung-Han Wu, Howin Jian Hing Ma, Paul Baessler, Roxanne Kate Balanay, Tyler R. Ray

**Affiliations:** ^1^ Department of Mechanical Engineering, University of Hawaiʻi at Mānoa, Honolulu, HI 96822, USA.; ^2^ Department of Cell and Molecular Biology, John A. Burns School of Medicine, University of Hawaiʻi at Mānoa, Honolulu, HI 96813, USA.

## Abstract

Skin-interfaced wearable systems with integrated microfluidic structures and sensing capabilities offer powerful platforms for monitoring the signals arising from natural physiological processes. This paper introduces a set of strategies, processing approaches, and microfluidic designs that harness recent advances in additive manufacturing [three-dimensional (3D) printing] to establish a unique class of epidermal microfluidic (“epifluidic”) devices. A 3D printed epifluidic platform, called a “sweatainer,” demonstrates the potential of a true 3D design space for microfluidics through the fabrication of fluidic components with previously inaccessible complex architectures. These concepts support integration of colorimetric assays to facilitate in situ biomarker analysis operating in a mode analogous to traditional epifluidic systems. The sweatainer system enables a new mode of sweat collection, termed multidraw, which facilitates the collection of multiple, independent sweat samples for either on-body or external analysis. Field studies of the sweatainer system demonstrate the practical potential of these concepts.

## Teaser

3D-printing enables a new frontier for creating sophisticated wearable sweat sensors with unique modes for collecting sweat.

## INTRODUCTION

Eccrine sweat is an attractive class of biofluid suitable for the noninvasive monitoring of body chemistry. Sweat contains a rich composition of biomarkers relevant to physiological health status including electrolytes (*1*), metabolites (*2*–*4*), hormones (*5*, *6*), proteins (*7*), and exogenous agents (*8*). Studies demonstrate the intermittent or continuous assessment of these, and other sweat biomarkers offer time dynamic insight into the metabolic processes of the body relevant to applications ranging from athletic performance (*9*–*11*) to medical diagnostics (*2*, *12*–*14*).

Recent advances in soft microfluidics, sensing technologies, and electronics establish the foundations for a unique class of skin-like epidermal microfluidic (“epifluidic”) systems. Adapting concepts from traditional lab-on-chip technologies, these wearable microfluidic platforms comprise sophisticated networks of channels, valves, and reservoirs embedded in elastomeric substrates (*15*–*20*). The thin, flexible device construct facilitates a conformal, fluid-tight skin interface by virtue of skin-compatible adhesives to collect sweat directly from sweat glands. The integration of colorimetric, fluorometric, and electrochemical measurement techniques enable such platforms to measure sweat constituents in situ across a wide array of applications and environments (*21*).

Traditional approaches for sweat collection use absorbent pads (*22*) or microbore tubes (*23*) pressed against the epidermis by virtue of bands or straps to capture sweat as it emerges from the skin. Requiring trained personnel, special handling, and costly laboratory equipment, such methods are incompatible with real-time sweat analysis and prone to sample contamination or loss (*24*). Epifluidic devices eliminate external sample contamination by virtue of the intrinsic encapsulation of the microfluidic network and conformal skin interface. Such systems are vulnerable to surface contamination from exogenous agents present on the epidermis, such as cosmetics or natural oils, without careful preparation of the skin surface before device attachment. Furthermore, the dependence on an adhesive interface for skin attachment limits these devices to single-use applications. Upon removal, the risk of contamination, potential sample loss, and active sweat response of previously covered glands pose substantial challenges to reapplication and continued sweat collection.

The typical epifluidic fabrication pathway uses soft lithography techniques (*25*) to produce devices with microfluidic components and complex geometries. A common, well-established process for fabricating lab-on-chip microfluidic devices (*26*), soft lithography, requires high-precision molds to form discrete, patterned layers of an elastomeric material [e.g., poly(dimethylsiloxane) (PDMS)] that when bonded together yield a sealed device. Traditionally, producing molds with sufficient feature resolution (>20 μm) requires expensive, time-consuming processing methods [micromachining (*27*) and micromilling (*28*)] and access to specialized environments (cleanroom). Such requirements result in elongated device design cycles, inequitable access to equipment necessary for innovation, and additional challenges for commercial deployment due to incompatibilities with large-scale manufacturing.

Additive manufacturing (AM), or three-dimensional (3D) printing, represents an attractive alternative to conventional planar (2D) fabrication methods. AM offers powerful capabilities for producing structurally complex objects with true 3D architectures through a rapidly expanding library of printing methods. In general, these methods create solid objects in a sequential, layer-by-layer manner directly from a digital computer-aided design (CAD) file. In the context of microfluidics, the use of 3D printing is well established (*29*) for the rapid, cost-effective fabrication of high-resolution templates for soft lithography. In particular, vat photopolymerization techniques [e.g., resin-based printing, stereolithography, digital light processing (DLP), and continuous liquid interface polymerization] (*30*) enable rapid production of microscale features (>100 μm) over large areas (>600 mm^2^) with high precision (*31*). Innovations in printer hardware, software processing, and materials chemistry further extend these 3D printing capabilities to enable the direct production of enclosed microfluidic channels for lab-on-chip applications. Although manufacturers advertise printers with high resolution (*xy* resolution: >50 μm and *z*-resolution: >5 μm), in practice, the obtainable channel dimensions and device complexity are typically limited to millifluidic features (>250 μm) (*29*). Printer specifications represent only one key constraint to printing devices with micron-scale internal fluidic features (<100 μm). Successful fabrication requires optimization of other critical factors including printing technology (e.g., vat photopolymerization versus extrusion), feature design and spatial location, and printer-dependent parameters. AM process optimization, particularly for vat photopolymerization, demands careful attention to the chemistry of printed materials (*30*, *32*). Resin formulations must simultaneously satisfy application specific requirements, such as biocompatibility or optical clarity, while preserving printability. Recent reports (*32*, *33*) leverage specialized DLP-based printers and customized resins to fabricate devices containing microfluidic components with <50-μm dimensions.

In general, wearable system designs must address the inherent mismatch between the mechanical properties of skin and rigid, planar device components. The most advanced platforms fabricated by conventional (non-AM) methods exploit sophisticated strategies, combining complex device geometries and soft (low modulus) materials to establish a seamless, nonirritating epidermal interface. Recent advances in soft materials chemistry support 3D printing approaches to fabricating wearable devices for applications spanning biophysical (*34*), biochemical (*35*, *36*), and environmental (*37*) monitoring. However, such capabilities remain limited for the 3D fabrication of epifluidic devices as a result of the high Young’s moduli of the primary material chemistries (i.e., methacrylate-based resins) (*38*) suitable for printing high-resolution microfluidics. Current efforts to fabricate skin-interfaced 3D printed microfluidics use alternative printing methods [e.g., fused deposition modeling (*34*) and direct ink writing (*39*)] that support fabrication with low modulus materials at the expense of printer resolution (>200 μm). In the context of epifluidics, the ideal fabrication scheme would use resin-based printing to fabricate devices with feature sizes comparable to conventional methods with biologically compliant form factors. Such an approach would transform the fluidic design space with truly 3D device architectures while enabling a rapid, iterative design process, facilitating individual-specific device customization, and reducing the cost for low-volume production.

This paper introduces a set of strategies, processing approaches, and microfluidic designs that support such fabrication capabilities using a commercial DLP 3D printer in a simple manner of operation. A modular 3D printed epifluidic platform, termed a “sweatainer,” demonstrates several unique aspects of an AM approach to fabricating epifluidic systems. This platform, to our knowledge, represents the first 3D printed epifluidic platform with true microfluidic dimensions. Specifically, the results highlight the potential of a true 3D design space for microfluidics through the fabrication of fluidic components (channels and valves) with previously inaccessible complex architectures. Printer optimization strategies and systematic experiments enable realization of micron-scale feature sizes (<100 μm) and enhancement of optical transparency of 3D printed channels. In combination, these concepts support integration of colorimetric assays to facilitate in situ biomarker analysis operating in a mode analogous to traditional epifluidic systems. Drawing inspiration from the vacutainer blood collection tube, the sweatainer system introduces a novel mode of sweat collection, termed “multidraw.” This method overcomes the inherent limitations of single-use devices by enabling the collection of multiple, independent pristine sweat samples during a single collection period. Field studies of the sweatainer system demonstrate the practical potential of these concepts.

## RESULTS

### Sweatainer system design

**Fig. 1.**
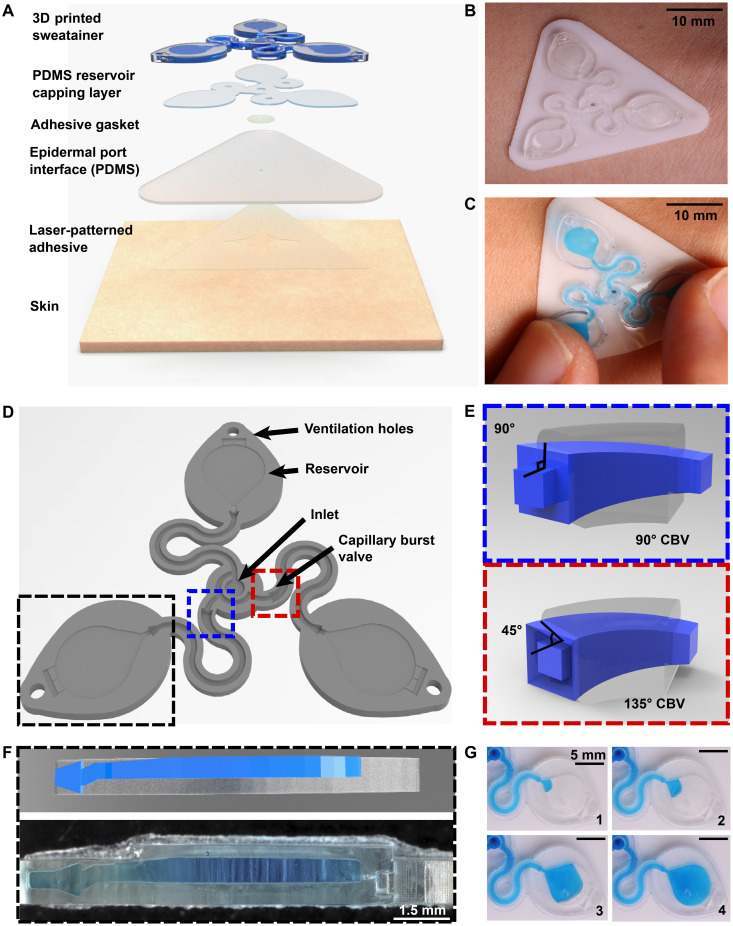
Schematic illustrations and optical images of the 3D printed epidermal microfluidic devices for the collection and analysis of sweat. (**A**) An exploded render highlights key components of the sweatainer system and epidermal interface (port). PDMS, poly(dimethylsiloxane). (**B**) A photograph of the sweatainer mounted on the ventral forearm of an individual before the onset of sweat collection. (**C**) The construct of the sweatainer eliminates uncontrolled fluid transport under mechanical loading (e.g., finger pressure and device removal). (**D**) Illustration of the sweatainer highlighting key device aspects including the inlet, capillary burst valves (CBVs; blue and red dashed area), collection reservoir, and ventilation holes to eliminate backpressure. (**E**) Renders of three-dimensional (3D) CBV designs enabled by 3D printing with diverging angles of 90° (top) and 135° (bottom). (**F**) 3D printing enables fabrication of device geometries in a true 3D space as shown by the computer-aided design (CAD) render (top) and photograph of actual device (bottom). Location of sweat appears in blue. (**G**) Photographic sequence highlighting the complete filling of a sweat collection reservoir.

Figure 1A shows a schematic illustration of the two primary modules of the sweatainer system: (i) the sweatainer device and (ii) an epidermal port interface. The sweatainer consists of a 3D printed microfluidic network of enclosed channels and unsealed reservoirs, a reservoir capping layer of PDMS (thickness: 200 μm), and a gasket formed from ultrathin biomedical adhesive (3M 1524; thickness: 60 μm). The bonded 3D printed photocurable resin structure and PDMS capping layer, as presented in Materials and Methods, define a closed microfluidic structure. Introduction of either dye or colorimetric assay before bonding enables sweat visualization or chloride concentration analysis, respectively. The cross-sectional width and thickness of the filleted serpentine channels presented here are 1200 and 1000 μm, respectively. The width and height of the rectangular-shaped internal microfluidic channels are 600 and 400 μm, respectively. The filamentary design of the rigid 3D printed structure (Young’s modulus: ~975 MPa) follows from the well-established principles of stretchable electronics (*40*) to impart sufficient stretchability to form a mechanically robust conformal interface. The gasket establishes a temporary, fluid-tight seal with the epidermal port interface permitting facile sweatainer application and removal via reversible adhesion to the PDMS surface.

The epidermal port interface comprises a thin film of pigmented PDMS (white, thickness: 400 μm) and a medical-grade adhesive layer (3M 1524) with laser-patterned openings. The adhesive layer facilitates a biocompatible, fluid-tight interface with the epidermis in which the patterned opening defines the sweat collection region (~180 mm^2^). An aligned access point on the backside of the sweatainer allows sweat to enter the system directly from the skin with flow driven by the natural pressures created by the sweat glands. The sweatainer design can support collection of 50.8 μl of sweat (10.8 μl per reservoir and 18.4 μl of channel network). A fully assembled representative system appears in Fig. 1B, where it is shown worn on the ventral forearm. Figure 1C demonstrates the insensitivity of the sweatainer to mechanical deformation through the absence of uncontrolled fluid flow during physical handling (finger pressure). The schematic illustration in Fig. 1D shows the microfluidic network within the 3D printed sweatainer. Sweat enters the device by the central inlet and flows through a microfluidic channel leading to a series of capillary burst valves (CBVs) and corresponding reservoirs. The CBV at the ingress of each reservoir permits fluid flow only after exceeding a set pressure, thereby enabling time-sequential sweat collection (*20*). Integrated ventilation holes (width: 100 μm and height: 200 μm) on the reservoir eliminate the backpressure that would evolve from trapped air and impede flow. The high-barrier properties of the photocurable resin support a low sweat evaporation rate with minimal mass loss over a 24-hour period (fig. S1 and table S1).

A key feature of this system is the use of AM to enable fully 3D, monolithic microfluidic designs comprising sophisticated nonplanar internal channel structures, spatially graded geometries, and 3D CBVs. Representative examples of 3D CBVs and the spatially graded, nonplanar geometries enabled by this fabrication method appear in Fig. 1 (E and F, respectively). By comparison, soft lithography fabrication methods restrict the design space of traditional lab-on-chip and epifluidic devices to planar (2D) channel configurations. Although lamination of multiple channel layers can yield elaborate 3D microfluidic networks, each component layer is inherently a planar geometry. As detailed in the sections that follow, the 3D fabrication expands the design space for CBVs with finer control over resultant burst pressure in comparison to planar CBVs. In a similar manner, spatially graded geometries improve sweat collection efficiency by permitting a continuous transition between the microfluidic channel and reservoir (Fig. 1F). This engineered interface, in combination with ventilation holes, ensures a uniform fluid front during reservoir filling (Fig. 1G, blue dye for visualization), thereby eliminating trapped air bubbles that result from a rapid expansion.

### Design and DLP printing considerations for optimized fabrication of 3D printed epifluidic devices

Successful fabrication of a fully enclosed microfluidic channel with feature sizes at the *xy* plane resolution limit of current DLP printers (~30 to 50 μm) depends on several related factors including: design aspects (e.g., channel vertical position), print process parameters [e.g., layer height, layer cure time (LCT), and print speed], and printer hardware (e.g., projector light power and wavelength). Optimization of user-adjustable factors results in a robust print process suitable for producing microfluidic devices with sufficient optical clarity, dimensional fidelity, and mechanical performance for use in epifluidic applications.

**Fig. 2.**
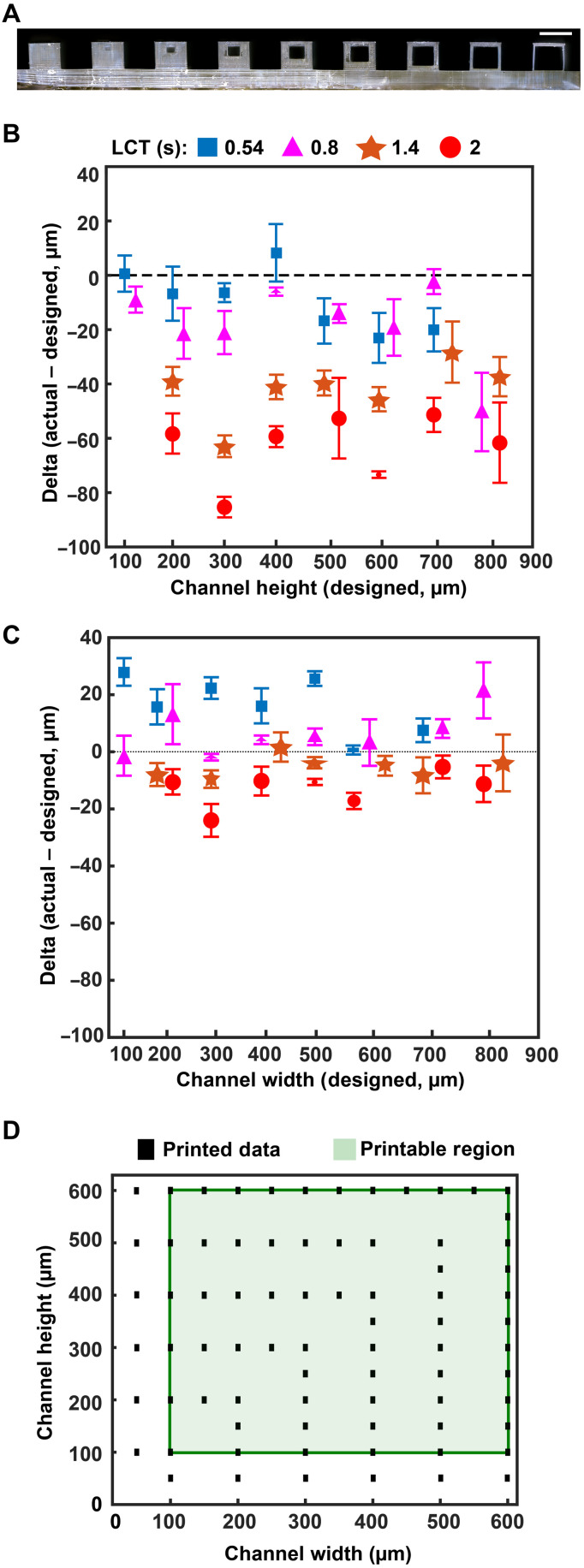
Optimized design strategy for fabricating 3D printed epifluidic devices with prescribed channel geometries. (**A**) Photograph of 3D printed test channels [100 to 900 μm, square; 2-s layer cure time (LCT)]. (**B**) Plot of variation of printed channel height from designed dimensions as a function of LCT. (**C**) Plot of variation of printed channel width from designed dimensions as a function of LCT. (**D**) Plot highlighting the printable region of the digital light processing (DLP) printer used in this work for various channel dimensions relevant to epifluidic devices.

As expected, epifluidic device performance is dependent on the dimensional accuracy of a fabrication process. If not quantified, then unintended deviation from designed dimensions can adversely affect component performance (i.e., CBV burst pressure) or measurement accuracy (i.e., sweat volume, sweat rate). Fabrication of test structures (Fig. 2A) comprising a sequence of square channels (width and height range: 100 to 900 μm, 100-μm increments; length: 5 mm) embedded in a square base (width and height: 1 mm) facilitate determination of the minimum printable channel dimensions and sidewall thickness (minimum of 50 μm). The asymmetric vertical position of the channels establishes a uniform capping layer (100 μm) across all dimensions tested. Because the DLP printer fabricates the structure in an inverse manner (Fig. 2A, base prints first), the channel position minimizes photopolymerizing resin trapped in the channel during the printing process.

Experimental studies reveal the similarly strong influence of LCT on print success and device quality. The LCT defines the energy dose used to cross-link the photopolymer given in time (seconds). The projector wavelength is hardware defined (385 nm for this work), and varying the power is not typically user accessible. Systematic studies of four LCT settings—selected starting from the minimum (0.54 s) to maximum values (2.0 s; 0.6-s interval) beyond which channels could not be fabricated successfully—establish a relationship among print performance (i.e., channel printed successfully), dimensional accuracy, and optical clarity. Measurement results from optical microscope images, shown in Fig. 2B for channel height and Fig. 2C for channel width, highlight the relationship between LCT and printed channel dimensions. The proportional relationship between increasing LCT and light propagation into the *z* dimension (thickness) of the masked regions (i.e., channels) results in smaller than designed channel heights. By comparison, the dimensional accuracy for a given channel width depends primarily on the size of the DLP printer pixels (*xy* plane resolution) rather than LCT. The observed positive channel width variation with decreasing LCT indicates incomplete photopolymerization. Subsequent postprocessing removal of uncured resin yields channels with dimensions greater than designed. In combination, these results establish the printable region for an epifluidic design as a function of LCT. As shown in Fig. 2 (B and C), successful fabrication of a 100-μm square channel requires a short LCT (i.e., 0.54 and 0.8 s), whereas a longer LCT results in photopolymerization of the otherwise unreacted resin. Conversely, for large dimensions (>700 μm square channels), a short LCT produces channels too fragile to survive printing and postprocessing due to incomplete photopolymerization. These results establish an LCT of 0.8 s as the optimal setting for balancing printability with dimensional accuracy for the printed epifluidic devices described in subsequent sections.

Additional systematic experiments establish the DLP-printable design space for epifluidic-relevant dimensions (100 to 600 μm). Evaluation of print success as a function of channel dimensions (width and height) for an enclosed microfluidic channel (length: 30 mm) identifies the printable region (Fig. 2D). An encapsulated microfluidic channel capable of supporting unrestricted fluid flow, in contrast to a sealed or partially restricted channel, defines a successful print. Intuitively, print failure rate increases as the enclosed channel dimensions approach the printer *xy* plane resolution limit (~32-μm x 32-µm square pixel). Results show a channel dimension of 100 μm (either width or height) corresponds to the lower limit for a successful printed device.

### Print process optimization to support colorimetric analysis in 3D printed epifluidic systems

The optical transparency of a 3D printed microfluidic device depends on several factors including material selection, printer hardware (e.g., build plate and vat surface material), postprocessing, and surface roughness. In contrast to the typical surface roughness feature size necessary for optical transparency (<10 nm) (*41*), DLP printers produce parts with microscale surface roughness, resulting in a semi-translucent appearance (*32*).

**Fig. 3.**
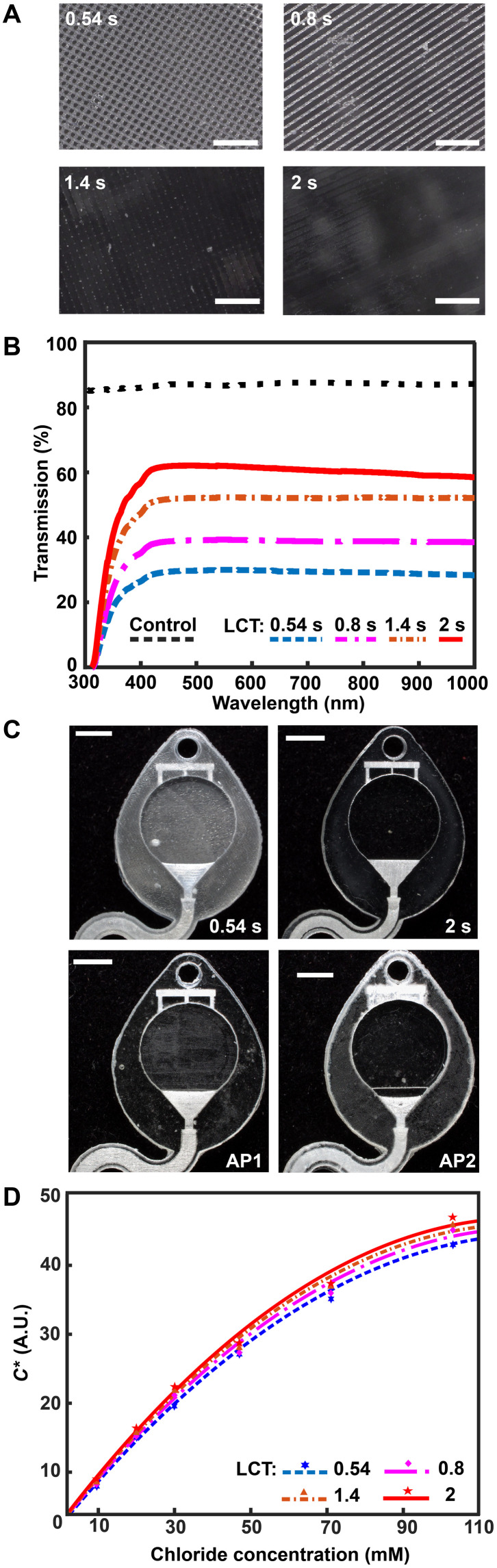
Optimized design strategy for enabling colorimetric analysis in 3D-printed epifluidic systems. (**A**) Optical micrographs of the surface of parts printed with different LCT settings. (**B**) Plot of light transmission of commercial and resin-printed cuvettes measured with ultraviolet-visible (UV-Vis) spectrometer. (**C**) Photographs of epifluidic reservoirs fabricated using static (0.54 s, 2-s LCT) and adaptive (AP1 and AP2) printing processes illustrating differences in optical transparency. (**D**) Calibration curves as a function of LCT highlighting improvement in optical transparency (and thus colorimetric performance) with increasing LCT. A.U., arbitrary units.

As mentioned previously, the digital micromirror device (DMD) pixel size governs the *xy* plane resolution of a DLP printer. Minute gaps between individual DMD elements locally reduce reflected light intensity, yielding a surface roughness with features corresponding to DMD pixel size and layer height. While specialized printing methods (grayscale) (*42*) or printer hardware (oscillating lenses) (*43*) offer sophisticated strategies to reduce aliasing and improve surface roughness, the fundamental approach to eliminating this defect mode is enhancing the uniformity of projected light to ensure complete photopolymerization. Figure 3A illustrates that increasing the exposure dose by lengthening the LCT eliminates the observed grid pattern defects (from DMD element gaps) and improves optical transparency. Ultraviolet-visible (UV-Vis) spectroscopy experiments examine the transmission properties of 3D printed microcuvettes in comparison to a commercial plastic cuvette (Fig. 3B). While results show substantial modulation of light transmission with increasing LCT, ranging from ~20 (LCT: 0.54 s) to ~60% (LCT: 2 s), the reference commercial plastic cuvette offers higher light transmission (~80%). Intuitively, there is no observed wavelength dependence for light transmission within the Vis spectrum (400 to 1100 nm) beyond the anticipated strong absorbance within the UV region (<400 nm, necessary for photopolymerization) for the 3D printed samples. As a consequence of the presence of both the UV absorber and photoinitiator in the resin, green parts (i.e., before curing) have a light yellow hue. As presented in Materials and Methods, completion of the postprocessing sequence eliminates part coloring (fig. S2).

In addition to LCT, layer height affects both overall device quality (e.g., vertical resolution, optical clarity, and channel roughness) and print time, which corresponds to device yield. Conventional approaches to vat photopolymerization use constant values for a given print run (i.e., fixed layer height and LCT). At present, only one manufacturer [Formlabs (*44*)] supports an adaptive layer height process to increase print speed by adjusting layer height as a function of model detail (i.e., small layers for fine features and thick layers for coarse features). Adaptive printing is an attractive process for obtaining expanded design flexibility for 3D printed epifluidic systems. Although not supported by default, a combination of custom software and manual geometric code programming in this work enables definition of both layer height and LCT as a function of model dimensions. The representative example shown in fig. S3 illustrates the capabilities of this adaptive printing process to fabricate a cube (all dimensions: 2 mm) using four layer heights (5, 10, 30, and 50 μm) in an arbitrary order. By comparison to a constant LCT and layer height setting printing process, this approach enables successful, time-efficient fabrication of epifluidic systems with complex geometries and superior device quality.

Colorimetric assays facilitate passive, battery-free in situ quantitative measurement of sweat biomarkers. A chemical reagent reacts with a target species to generate an optical signal proportional to analyte concentration (*45*). Accurate colorimetric analysis requires channels with uniform height (i.e., path length), a high degree of optical transparency, and integrated color reference markers to support reliable image processing under variable ambient lighting conditions (*46*). The layer-by-layer control over LCT and layer height parameters enabled by an adaptive printing process is critical for fabricating microfluidic devices with the requisite surface finish and optical transparency to support colorimetric analysis. Figure 3C illustrates the influence of an adaptive LCT print process on the optical transparency of microfluidic channels. The optical clarity for two representative sweatainer reservoirs manufactured using a layer-constant LCT (0.54 and 2 s) increases with longer LCT (Fig 3C). While beneficial for reducing nonuniform illumination, the increased UV dose results in undesirable curing of resin in enclosed features (channels and CBVs). By comparison, an adaptive printing process (AP 1) using an LCT of 0.54 s for the reservoir surface and an LCT of 2 s for subsequent layers facilitates fabrication of a sweatainer with a translucent imaging plane, a transparent device, and preservation of internal channel features. An inverse adaptive printing process (AP 2; base LCT: 2 s and subsequent layer LCT: 0.54 s) results in an optically transparent imaging plane and a translucent device.

Systematic benchtop experiments evaluate the suitability of devices fabricated by adaptive printing for colorimetric analysis. The colorimetric assay silver chloranilate produces a dark violet color response proportional to chloride concentration. Imaging the device with a smartphone camera enables color extraction and subsequent quantification of color response. The inclusion of a color balance chart facilitates color calibration for each image. As in previous reports (*47*, *48*), converting images from native red, green, blue (RGB) color space to CIELAB color space—which expresses color as lightness (*L*), amount of green to red (a^*^), and amount of yellow to blue (b^*^)—ensures device-independent color sampling. Conversion of the a* and b* components to chroma (C^*^) by the relation

$$C^{*}={\left({a^{*}}^{2}+{b^{*}}^{2}\right)}^{1/2}$$ (1)

yields a calibration curve with chloride concentration by a power-law relation (fig. S4). Figure 3D shows calibration charts created from 3D printed sweatainers with different LCT parameters and reference colorimetric assay solutions. This plot reveals that the improvement in optical clarity with increasing LCT provides a corresponding enhancement in the range of detectable color measurements. As these findings indicate, an adaptive printing process is essential for fabricating epifluidic devices with an optical transparency sufficient to support colorimetric analysis.

### 3D CBV designs for sequential sweat analysis

CBVs are a key component for the sequential analysis of sweat biomarkers in many epifluidic platforms. The time dynamic variations in sweat rate arising from physical (e.g., sweat gland density), physiological (e.g., exertion and emotion), and external factors (e.g., temperature and pH) result in corresponding changes in analyte concentration. As previously described, CBVs prevent flow for fluid pressure conditions below a designed threshold [bursting pressure (BP)]; when the fluid pressure exceeds the BP, the CBV immediately bursts. Operating without use of actuation or moving components, CBV BP is governed by valve geometry.

The Young-Laplace equation describes the BP for a CBV (rectangular channel) as (*49*)

$$\mathrm{BP}=-2\sigma \left[\frac{\cos\theta_{I}^{*}}{b}+\frac{\cos\theta_{A}}{h}\right]$$ (2)

where σ is the fluid surface tension, θ_A_ is the critical advancing contact angle for the channel (material dependent, θ_A_ = 120° for PDMS) (*50*), $\theta_{I}^{*}$ is the minimum of either θ_A_ + β or 180°, β is the channel diverging angle, and *b* and *h* are the diverging channel width and height, respectively. As the second term of Eq. 2 is constant for a planar (2D) CBV, channel width and diverging angle govern the BP for a given CBV. In practice, epifluidic device designs use geometric restrictions (i.e., modifications to channel width) to control valve BP.

**Fig. 4.**
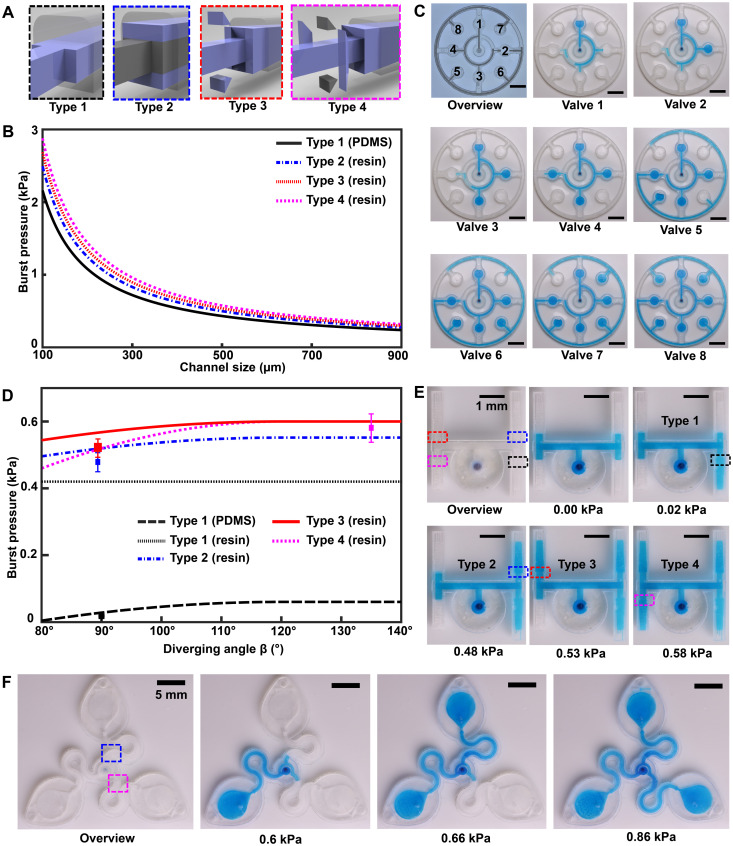
3D CBV designs for sequential sweat analysis. (**A**) Schematic renders highlighting four design types of CBVs used in this work. Areas highlighted in blue indicate differences between CBV designs. (**B**) Plot of the theoretical maximum bursting pressures (BPs) calculated from the Young-Laplace equation as a function of channel size for a square geometry. (**C**) Sequence of photographs illustrating the performance of different CBV designs (labels 1 to 8). Use of backside illumination for the overview photograph facilitates visualization of valves and channels. (**D**) A sequence of photographs shows a 3D printed H channel with one central inlet and four CBVs (color indicates CBV design and fixed channel geometry) filling sequentially, highlighting the fluid control enabled by a true 3D CBV. (**E**) Plot of the theoretical BP as a function of diverging angle β for a channel with a fixed geometry (width: 600 μm and height: 400 μm). The CBV designs are identical to (B). (**F**) A sequence of photographs highlighting performance of the 3D printed sweatainer design used in human participant testing.

The 3D printing concept for epifluidic devices presented here expands CBV capabilities by enabling a full 3D CBV design. As a consequence, Eq. 2 can be written as

$$\mathrm{BP}=-2\sigma \left[\frac{\cos\theta_{I}^{*}}{b}+\frac{\cos\theta_{J}^{*}}{h}\right]$$ (3)

for a 3D CBV, where $\theta_{J}^{*}$ is the minimum of either θ_A_ + γ or 180° and γ is the channel diverging angle (*z* axis). It follows that for a microfluidic channel with fixed dimensions, the CBV BP becomes a function of the channel diverging angles (β and γ). Computational predictions of four representative CBV designs, presented as a schematic in Fig. 4A with parameters specified in Table 1, illustrate this relationship. Figure 4B shows the theoretical BP versus channel size (square channel) for the four CBV designs with σ = 0.072 N/m (surface tension of water) and θ_A_ = 120° (PDMS) for the 2D CBV (type 1) and θ_A_ = 60° for the 3D CBVs (resin, types 2 to 4). It is shown that BP is inversely proportional to channel size. As expected, the analytical model reveals that for a given channel size BP increases for 3D CBV designs (resin) in comparison to a 2D CBV (PDMS). Within the subset of 3D CBV designs, the channel diverging angles (β and γ) dictate the valve BP (BP_Type4_ > BP_Type3_ > BP_Type2_).

**Table 1.** Diverging angle parameters for CBV type. CBV, capillary burst valve; 2D, two-dimensional; N/A, not applicable.

CBV type	Geometry	β	γ
1	2D	90°	N/A
2	3D	90°	90°
3	3D	90°	135°
4	3D	135°	135°

Benchtop experiments yield measurements of CBV BPs by means of a positive pressure displacement pump apparatus that perfuses water (dyed blue for visualization) into the microfluidic network at defined pressures. Figure 4C shows a representative test of the sequential filling performance of a network of 2D and 3D CBV-gated reservoirs, labeled chronologically in order of increasing BP. Table 2 and fig. S5 detail the CBV design parameters, theoretical CBV BPs, and effective theoretical BPs, which consider the theoretical CBV BP and fluidic resistance of the microfluidic channel network. Imperfections resulting from the 3D printing process result in experimental BP values below theoretical limits.

**Table 2.** Design parameters for CBVs. BP, bursting pressure.

Valve no.	CBV type	Channel width (μm)	Channel height (μm)	Theoretical CBV BP (kPa)	Effective theoretical BP (kPa)
1	N/A	600	600	N/A	0.003
2	2	600	600	0.42	0.421
3	2	500	500	0.50	0.509
4	1	200	600	0.50	0.515
5	2	500	500	0.50	0.517
6	3	500	500	0.54	0.567
7	4	500	500	0.58	0.618
8	4	400	400	0.72	0.773

The 3D design space provides attractive capabilities for fine-scale control over CBV performance to enable compact fluid control features within epifluidic devices. Varying the diverging angle design parameters (β and γ) for a 3D CBV results in substantial differences in BP for valves with similar dimensions and form factors. Systematic experiments performed in similar manner as described previously verify the correlation between diverging angle and BP for the 3D CBV architectures illustrated in Fig. 4A with identical channel dimensions. Figure 4D shows a representative test of 3D CBV performance via a 3D printed microfluidic device with channels arrayed in an H configuration (channel dimensions: 600-μm width and 400-μm height). As Fig. 4E highlights, BP increases with β for a resin-based 3D CBV in contrast to a PDMS-based 2D CBV baseline reference. Material properties limit the valve design space on account of the BP dependence on contact angle. For hydrophobic materials such as PDMS (i.e., contact angle >120°), β values greater than 60° reduce to 180°, resulting in BP value dependent only on channel width (*b*). By comparison, the expanded design range, in which β governs valve BP, results from the smaller contact angle of hydrophilic materials (i.e., resin). The experimental results support these trends predicted by the analytical model with the variation between measured and predicted values attributed to geometric imperfections inherent to the fabrication process (i.e., slight rounding of corners) (*51*). A similar trend occurs for valve designs in which γ varies with respect to a fixed β.

Additional studies demonstrate 3D CBV performance in a device architecture relevant to practical use. Robust operation requires CBV designs with BP within the physiologically relevant range of sweat secretory pressure (0.5 to 2 kPa) (*51*). Tests of the sweatainer design shown in Fig. 4F proceed in the same manner whereby water enters the device through a central inlet. Reservoirs fill sequentially in the order indicated as the CBVs at entrance of reservoirs no. 2 and no. 3 prevent fluid flow until reservoir no. 1 fills completely. Variation of CBV diverging angle defines the BP for CBV no. 1 (blue, 0.66 kPa) and CBV no. 2 (red, 0.86 kPa). These results validate the design of the sweatainer for use in on-body testing.

### Field studies of the sweatainer

**Fig. 5.**
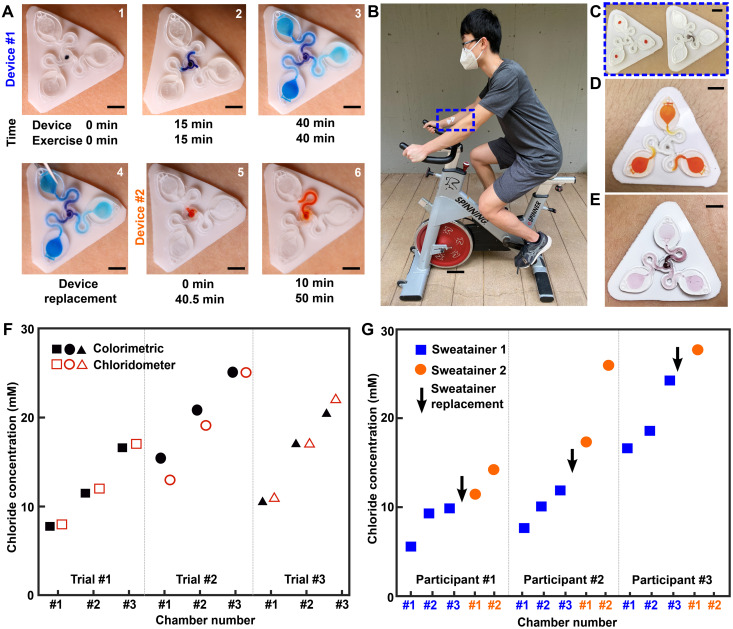
Sweatainer field studies. (**A**) Sequence of photographs highlighting operation of the sweatainer system. A sweatainer (device no. 1) collects sweat during an active exercise period, which, upon filling, is rapidly exchanged (<30 s) for a second sweatainer (device no. 2) facilitating multidraw sweat collection. (**B**) Photograph of sweatainer position during exercise trials (blue box). (**C**) A magnified view of the same sweatainer devices shown in (B) before the onset of sweating. The sweatainer shown on left is for collection (control) with the device on the right for colorimetric analysis. (**D** and **E**) respectively show the collection and colorimetric sweatainers at the conclusion of the exercise period. (**F**) Plot showing the concentration of sweat chloride from the collection (chloridometer) and colorimetric sweatainers for three independent exercise trials for a single participant (stationary cycling, 50 min, constant power). (**G**) Plot of showing sweat chloride concentration from two different colorimetric sweatainers worn sequentially (i.e., replaced during trial) during a predefined exercise period (stationary cycling, 50 min, constant power). The total sweat volume lost by a given individual during this exercise period corresponds to the total number of filled sweatainer chambers. Scale bars, 5 mm.

A pilot study comprising healthy adult volunteers (*N* = 8) exercising on a stationary bike explores the on-body performance of the sweatainer system. Following the protocol detailed in Materials and Methods, the sweatainer intimately couples to the ventral forearm of a participant by means of the epidermal port (PDMS/skin-safe adhesive). Participants cycled at moderate intensity for a period of 50 min under controlled environmental conditions [22°C, 59% relative humidity (RH)]. Upon entering the device from the skin, sweat proceeds to sequentially fill the microfluidic reservoirs. The addition of chloride-free dye at the sweatainer inlet aids in visualization. Periodic imaging with a smartphone camera during exercise facilitates monitoring fill performance. The sweatainer typically fills within 40 min from the initiation of exercise; after filling, the device is exchanged mid-exercise with a new sweatainer in a seamless manner. Figure 5A highlights this event sequence with sweatainers distinguished by distinct visualization dyes (device no. 1: blue and device no. 2: orange). The simplicity of the exchange facilitates a rapid replacement time (<30 s), thereby minimizing potential interruption to the sweat collection process. In all tests, the adhesive gasket maintains a robust, water-tight interface between the sweatainer and epidermal port evidenced by the absence of observed leaks. The 3D printed sweatainer resists mechanical deformation during detachment, thereby eliminating unconstrained fluid flow. In combination, these features support the multidraw collection of pristine sweat samples and reduce the risk of sample contamination during collection process.

A second set of exercise tests focuses on the in situ measurement of the concentration of sweat chloride by colorimetric analysis. A sweatainer configured with an integrated colorimetric assay (replacing visualization dye) enables measurement of chloride concentration in collected sweat during exercise. Figure 5B shows the sweatainer mounted on the ventral forearm of a volunteer participant. Simultaneous deployment of a collection sweatainer (orange dye alone) in close spatial proximity of a colorimetric sweatainer (Fig. 5C) facilitates comparison of colorimetric chloride measurements with gold standard clinical methods for chloride analysis (chloridometer). The collection sweatainer operates in a similar mode to microbore tubes (i.e., Macroduct) traditionally used in clinical settings for collecting sweat for chemical analysis. Representative photographs of the colorimetric and collection sweatainers at the conclusion of an exercise period appear in Fig. 5 (D and E, respectively). As shown in Fig. 5F, for a representative participant, chloride concentrations measured using colorimetric sweatainers correlate well for given individual (Fig. 5F reports three independent exercise trials), within experimental uncertainties, to values determined using coulometry and are within the normal physiological range (*48*). The chronological sampling capabilities of the sweatainer enable monitoring of the time dynamic variation of sweat biomarkers. Figure 5G demonstrates the multidraw sweatainer operation for three participants (field study data for remaining five participants shown in table S2) during a fixed exercise period (stationary cycling, 50 min, constant power). In both sets of trials, the observed increase in sweat chloride concentration during exercise is consistent with results from previous studies (*52*). Here, an inverse relationship exists between the sweat duct efficiency in reabsorbing chloride and rate of sweat loss, resulting in a corresponding increase in sweat chloride. Factors such as fitness level, training status, and heat acclimation affect this relationship for a given individual. These findings demonstrate the sweatainer system as a viable platform for colorimetric-based biomarker analysis with reported values comparable to established clinical methods.

## DISCUSSION

The sweatainer system reported here introduces an AM approach to fabricating epidermal microfluidic devices to collect and analyze sweat. AM enables true 3D design of microfluidic channels and fluid control components, such as valves, with architectures typically inaccessible to planar (2D) fabrication methods. The detailed characterization and optimization of print parameters provides a pathway to fabricate microfluidic devices with enhanced optical transparency and feature sizes below 100 μm. Field studies using stationary cycling provide a practical demonstration of key concepts of the sweatainer platform including multidraw sample collection scheme and in situ colorimetric analysis of chloride concentration. Future studies will seek to investigate the generalizability of the sweatainer platform beyond clinical applications to sweat collection during more vigorous and dynamic physical activities through the development of optimized designs capable of supporting a broader spectrum of physical exertion.

The sweatainer platform represents a pivotal advancement in the collection and analysis of sweat samples. Inspired by the versatility of the vacutainer for blood collection, the sweatainer allows for the acquisition of multiple, independent aliquots of sweat from a single collection period. This collection mode enables an array of possibilities for sweat-based studies, including remote and at-home diagnostics, biobanking for future clinical research, and the integration of sweat analysis into existing clinical chemistry methods. Moreover, the utilization of AM for fabricating the sweatainer allows for customized geometries and streamlined integration into clinical workflows, further enhancing the potential of the platform for facilitating the quantification of ultralow concentration sweat biomarkers. The realization of multidraw sweat collection, enabled by the sophisticated sample collection strategies and customizable designs reported here, represents a major step forward in the field of sweat-based analysis.

## MATERIALS AND METHODS

### Fabrication of 3D printed epifluidic devices

Each epifluidic device design (3D) was created using CAD software (AutoCAD 2019, Autodesk, CA, USA). Subsequent export to a stereolithography file format (.stl) yielded a file suitable for direct use by the DLP resin printer (Prime 110, 385 nm, MiiCraft, Taiwan and Creative CADworks, ON, Canada). The included printer control software (Utility, version 6.3.0.t3) provided direct control over print parameters for each file including layer height (5 to 50 μm), dose, and lamp power. High-fidelity printing was achieved by application of a removable Kapton polyimide tape over the surface of the polished aluminum build plate. The applied tape was free of bubbles/wrinkles to ensure a smooth build surface free of defects.

Devices were printed using transparent resin (MiiCraft BV-007A, Creative CADworks, ON, Canada) and a 10-μm layer height (six devices per build plate, ~20 min total print time). Gentle removal of printed parts from the build plate, soaking in 1% detergent solution (Alconox-1232-1, Alconox, NY, USA) under sonication (CPX2800, Fisher Scientific, PA, USA) for 10 min, drying of device using clean dry air (CDA), postprint UV cure for 10 min (CureZone, MiiCraft, Taiwan), and postcure bake at 70°C for 30 min (Model 40E Lab Oven, Quincy Lab Inc., IL, USA) yielded a 3D printed epifluidic device suitable for direct use or integration with PDMS.

A three-step process (fig. S6) facilitated printing fully enclosed 3D printed devices. Printing epifluidic devices with open reservoirs (step 1) and postprint removal of uncured liquid resin by CDA (step 2) enabled enclosure of the devices with a thin capping layer (30 μm) by means of a second print process (step 3). The printed device remains fixed to the build plate during the printing process to ensure feature alignment. Following the previously described postprocessing steps yielded a fully enclosed epifluidic device.

### Fabrication of ultrathin capping layer for microfluidic channels in hybrid devices

Pouring liquid PDMS (10:1 base:curing agent; Sylgard 184, Dow Inc., MI, USA) with white pigment (3% w/w; Ignite White, Smooth-On Inc., PA, USA) onto a sacrificial mylar film (2 mil thickness), spin coating for 30 s [400 revolutions per minute (rpm) for reservoir capping layer and 200 rpm for epidermal interface layer], and curing in an oven (70°C, 2 hours) formed films with thicknesses of 200 and 400 μm, respectively. A CO_2_ laser cutter (30 W Epilog Mini 24, Epilog Laser, CO, USA) patterned the PDMS films into the final geometries used in the epifluidic devices. A medical-grade adhesive (1524, 3M Inc., MN, USA) is patterned in the same manner and bonded to the PDMS interfacial layer, established the epidermal interface for the device.

Hybrid 3D printed epifluidic devices use bonded PDMS capping layers to enclose 3D printed microfluidic reservoirs. Modification of a previously reported method (*53*) facilitated a strong bond between PDMS and the printed device. Specifically, rinsing with isopropyl alcohol (2-propanol, A416, Fisher Scientific, MA, USA), soaking in deionized (DI) water (Direct-Q 3 UV Water Purification System, MilliporeSigma, MO, USA) for 30 min, corona treating with air plasma (BD-20, Electro-Technic, IL, USA) for 30 s followed by immediate immersion in a 12% v/v solution of (3-aminopropyl)triethoxysilane (APTES; 440140, MilliporeSigma, MO, USA) for at least 20 min, rinsing in DI water, and drying with CDA prepared the oven-baked 3D printed device for bonding to PDMS. Pipetting colorimetric reagents or flow visualization dye (Soft Gel Paste, AmeriColor Corp., CA, USA) into predetermined regions occurred before sealing of the 3D printed device. After a 30-s corona treatment, laminating the PDMS capping layer to the APTES-modified printed surface sealed the epifluidic device. Heat treating the assembled device on a hotplate (70°C) under applied weight (3 kg) for 30 min formed a permanent bond. Removal of the sacrificial mylar layer, release from excess PDMS via laser cutting, and opening the central sweat ingress points using a 1.5-mm diameter circular punch (reusable biopsy punch, World Precision Instruments) yielded a final hybrid epifluidic device.

### Measurement of evaporation rate for 3D printed microfluidic networks

3D printed microfluidic devices (*N* = 7) with theoretical capacity (~101 ml) facilitated the measurement of the rate of evaporation. Sealing of the inlet and outlet of a device with parafilm after filling with DI water (dyed blue for visualization) formed the complete device for testing. Measurement of the initial sealed device mass (inclusive of water, film, and printed microfluidic system) using a microbalance (Sartorius Quintix 224-1S, Germany) enabled recording of mass loss at 2 and 24 hours. Devices were maintained at room temperature in a controlled laboratory environment reflective of anticipated use environment (22°C, 55% RH). An optical camera (Canon 90D, Canon EF 100 mm f/2.8L USM lens) facilitated observation of visual changes to fluid levels at each measurement interval.

### Characterization of 3D CBVs

A digital microscope (VHX-7100, Keyence Corp., Japan) produced micrographs of the devices. An optical camera (Canon 90D, Canon EF 100 mm f/2.8L USM lens) provided video capture capabilities (30 frames per second) for device analysis. Measurement of the CBV burst pressure consisted of a “fill test” in which water (dyed blue for visualization) entered a device until flow stopped the CBV. A modular, calibrated pressure displacement flow system (Flow EZ, Fluigent, France) controlled the fluid pressure and permitted near-instantaneous stepwise increase in pressure (0.1-mbar interval, 10-s dwell time). Video observation identified the pressure threshold for fluid to burst the valve.

### Measurements of transmission properties of 3D printed devices

A UV-Vis spectrophotometer (UV-1900i, Shimadzu, Japan) enabled quantification of the optical transmission properties of the printed devices (300 to 1000 nm, 0.5-nm interval). A commercial plastic cuvette (path length: 10 mm; Shimadzu) served as a reference (control). Four sets of 3D printed cuvettes (*N* = 3 per set) using a different LCT setting (0.54, 0.8, 1.4, and 2 s) enable quantification of the relationship between LCT and optical transmission (dimensions: height, 50 mm; width, 8 mm; path length, 1 mm; and volume, ~21 μl).

### Colorimetric assay for chloride

The chloride colorimetric assay solution resulted from thoroughly vortexing 50 mg of silver chloranilate (MP Biomedicals, CA, USA) in 200 μl of a solution of 2% (w/v) polyhydroxyethylmethacrylate (529265, MilliporeSigma, MO, USA) in methanol (A412, Fisher Scientific, MA, USA) to yield a homogenous suspension. Spotting 2 μl of this solution via laboratory pipette onto the 3D printed device near the central sweat ingress point, followed by drying in an oven for 30 min before encapsulation, prepared the epifluidic device for colorimetric chloride measurements.

### Standard color development and color reference marker preparation

Mixing sodium chloride (S271, Fisher Scientific, MA, USA) in DI water produced standard test solutions (0, 10, 20, 30, 50, 75, 90, 110, 130, and 150 mM). Clinical-grade chloridometer measurements (ChloroChek, ELITech Group Inc.) yielded validated test solution concentrations. Digital imaging and analysis of sample reservoirs (*N* = 7) containing one standard solution reacted with the silver chloranilate assay under uniform illumination formed a set of reference images. The sample reservoirs were of the same depth as the epifluidic channels to ensure accurate color representation.

### Digital image analysis for the evaluation of sweat chloride concentrations

A smartphone camera (iPhone 11 Pro Max, Apple, CA, USA) captured images during on-body field tests. A color calibration card (ColorChecker Classic, X-Rite, MI, USA) in the frame of each image facilitated accurate color extraction under various illumination conditions. An open-source photography software package (Darktable 3.0.0, Darktable.org) served as the platform for calibrating images using the color reference card. Analysis of calibrated images using MATLAB (R2019b, MathWorks Inc., MA, USA) enabled cropping multiple regions of interest (*N* = 3) from images and extraction of CIELAB color values (*L*, a*, and b*) for chroma analysis. Mapping of chroma values from colorimetric samples of known reference chloride solutions yielded colorimetric calibration charts with a power-law relationship. This calibration chart supported quantification of the sweat chloride concentration in on-body field testing.

### Human participant sweat analysis

The purpose of this pilot study was to evaluate the performance of the 3D printed epifluidic device and use in collecting and analyzing sweat. Testing involved healthy young adults (*N* = 8, six male and two female) as volunteers during normal physical activity (stationary cycling) with no additional human participant risk. The study was International Review Board (IRB) approved through the University of Hawaiʻi (IRB no. 2018-1440). Informed consent was obtained after explanation of the nature and possible consequences of study participation.

Cleaning of the forearm of each individual with an alcohol wipe prepared the skin for robust adhesion to the device. The exercise regime comprised stationary cycling under approximately constant working load for 50 min in a controlled laboratory environment (22°C, 55% RH).

Evaluation of the colorimetric sweatainer performance required individual participants (*N* = 3) to wear two separate sweatainers, one colorimetric and one collection (as a control), located in close proximity on the same arm. Before device removal, a photograph of the colorimetric sweatainer was recorded at the conclusion of the collection period for image processing and chloride analysis. Extraction of sweat from the individual reservoirs of the collection sweatainer at the conclusion of the exercise period facilitated chloride measurements using a ChloroChek Chloridometer.

Evaluation of sequential generation of aliquots of sweat required periodic monitoring the filling of the epifluidic device (*N* = 8). Once all reservoirs filled, as determined by visual observation, the initial device (attached at the start of the exercise period) was removed from the interfacial layer and replaced with a new device while simultaneously continuing to exercise.

## Notes

View/request a protocol for this paper from *Bio-protocol*.
