# Supplementary material for: Skin-interfaced microfluidic systems with spatially engineered 3D fluidics for sweat capture and analysis
Source: Sci Adv. 2023 May 3;9(18):eadg4272. doi: 10.1126/sciadv.adg4272 (PMC10881187; doi:10.1126/sciadv.adg4272)
Supplement: Supplementary file 1 — Figs. S1 to S6 Tables S1 and S2 [file sciadv.adg4272_sm.pdf]

Science Advances
AAAS

Supplementary Materials for

Skin-interfaced microfluidic systems with spatially engineered 3D fluidics for sweat capture and analysis

Chung-Han Wu *et al.*

Corresponding author: Tyler R. Ray, raytyler@hawaii.edu

Sci. Adv. **9**, eadg4272 (2023)
DOI: 10.1126/sciadv.adg4272

This PDF file includes:

Figs. S1 to S6
Tables S1 and S2

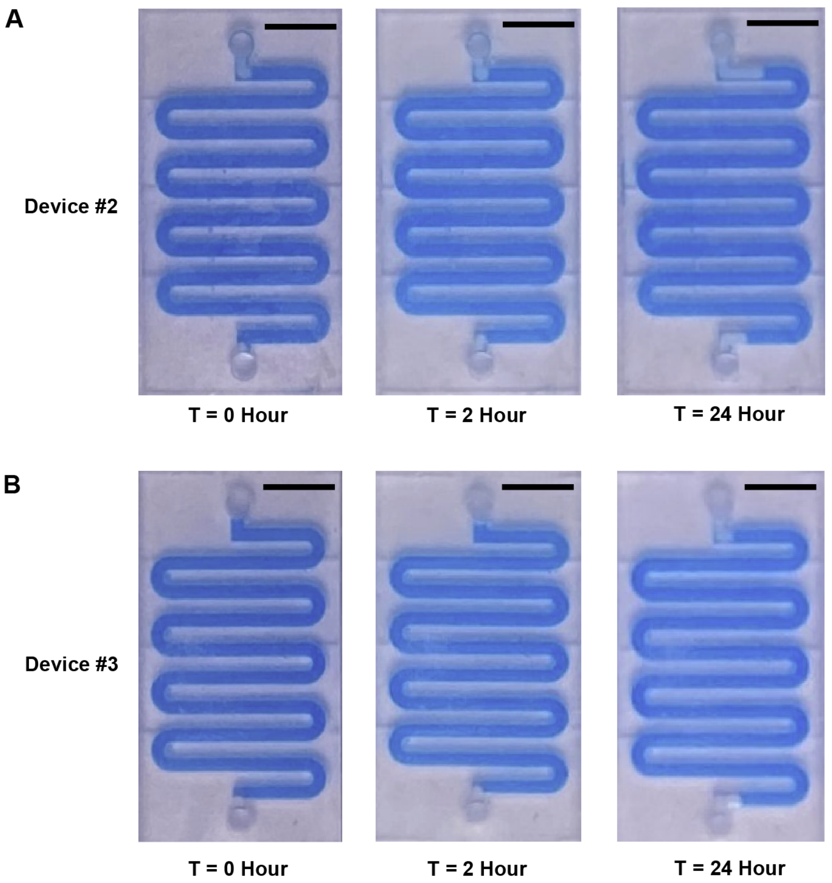

Figure S1. Evaporation measurement of sweat in 3D-printed microfluidic networks. Optical images of 3D-printed microfluidic devices used in evaporation measurements corresponding the highest (**A,** Device #2) and lowest (**B**, Device #3) evaporate rates recorded at 0, 2 and 24 h. Scale bars represent 5 mm.

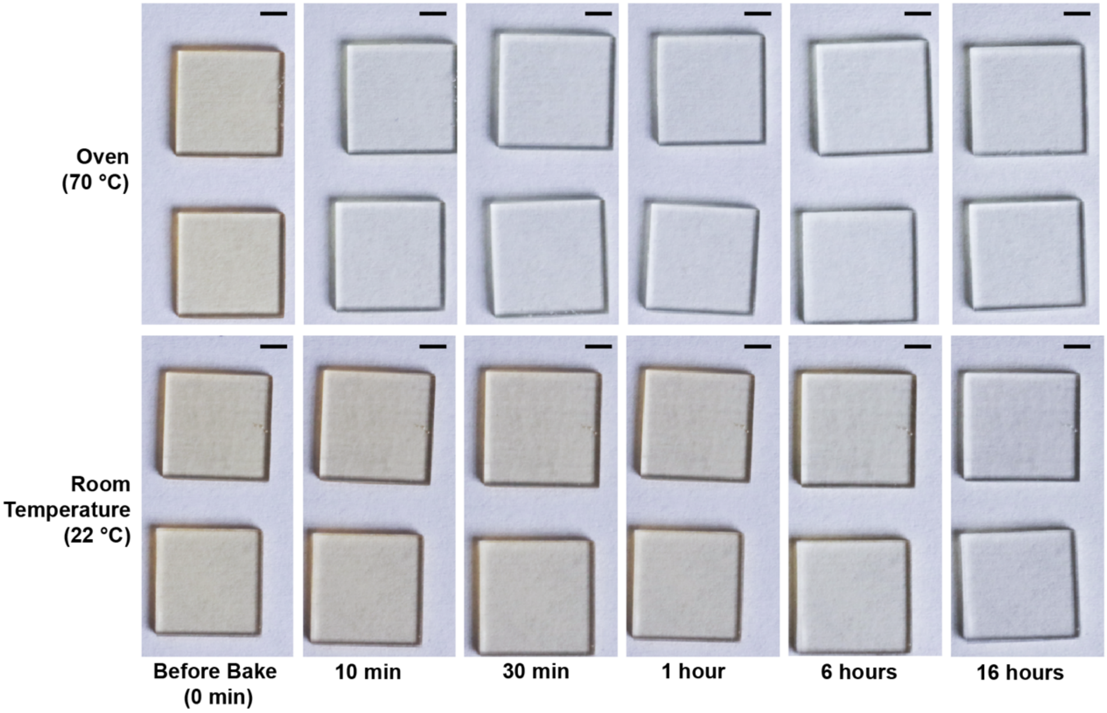

Figure S2. Influence of post-processing process on coloration of 3D-printed epifluidic devices. Optical photographs of printed parts imaged over a 16-hour timeframe under an elevated temperature (70 °C) bake with a room temperature (22 °C) cure as control. Images recorded under controlled lighting conditions and uniformly color corrected with the white background as the neutral reference point. Scale bars represent 2.5 mm.

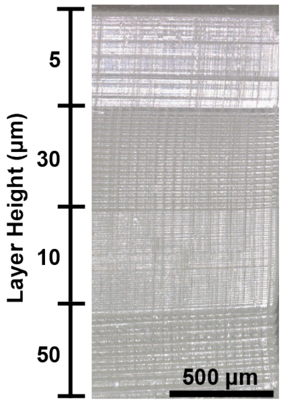

Figure S3. Adaptive printing enables layer-by-layer control over printing parameters. An optical image demonstrating the adaptive printing process. A 2 mm height cube is printed using 4 layer heights (5 μm, 10 μm, 30 μm, 50 μm) in an arbitrary, customized order.

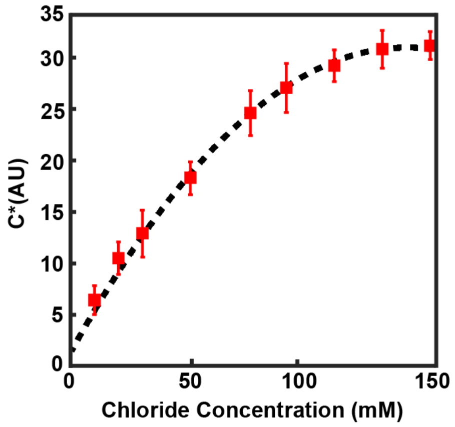

Figure S4. Calibration curve for colorimetric analysis of sweat chloride concentration. Chroma (C*) values for standard chloride solutions establish the calibration curve for colorimetric analysis of sweat chloride concentration as a function of extracted color from an optical photograph.

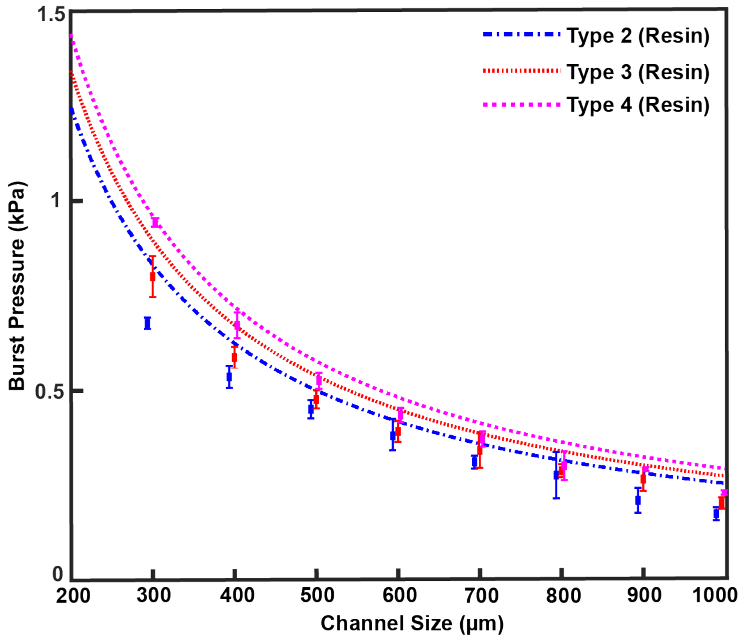

Figure S5. Burst pressures for CBV-gated reservoirs. Comparison of experimental BP values from benchtop experiments utilizing a pressure-based flow control apparatus for CBV valve designs (Type 2, 3, 4) as a function of channel size (300 – 1000 μm) with theoretical BP values.

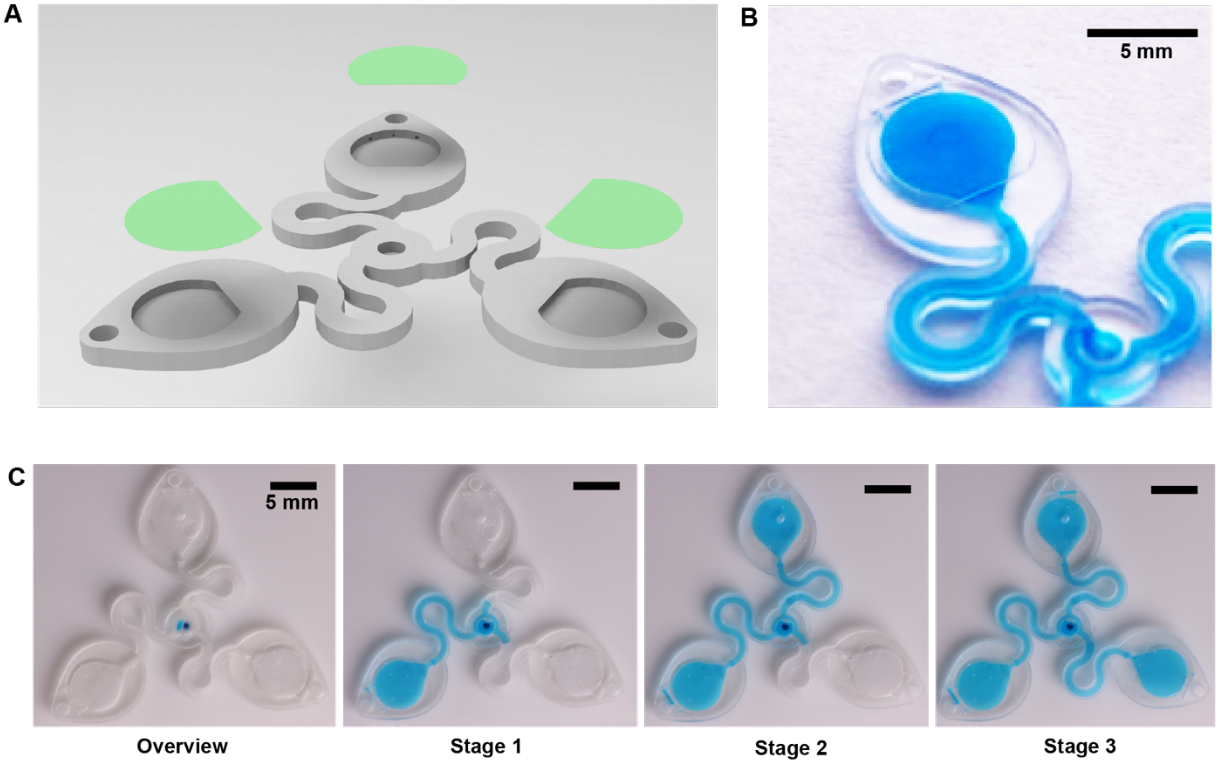

Figure S6. Fully enclosed 3D-printed sweatainer. Schematic illustrations and optical images of a 3D-printed sweatainer manufactured by a three-step print-pause-print process. (A) An exploded view of the printed sweatainer illustrating the two print processes (first print is gray, second print is green). B. Photograph of one reservoir after the second (encapsulation) print. C. Sequence of photographs demonstrating CBV performance as indicated by discrete filling steps (Stage 1 – 3).

Supplementary Table S1. 3D-printed microfluidic devices were loaded with blue-dyed water for evaporation measurement resulting from measurement of total mass (N=7 devices) at 0, 2 and 24 h. The averaged evaporation rate was computed based on 24 h evaporated mass. The photographs of the most (Device #2) and the least (Device #3) evaporation rates appear in Fig. S1.

Device #	Initial Mass (mg)	Mass After 2 Hour (mg)	Mass After 24 Hour (mg)	24 Hour Evaporated Mass (mg)	Averaged Evaporation Rate (mg/hour)
1	1114.5	1114.2	1113.7	0.8	0.03
2	1103.9	1103.3	1102.5	1.4	0.06
3	1101.7	1101.6	1101.6	0.1	0.00
4	1117.2	1116.9	1116.8	0.4	0.02
5	1103.1	1102.8	1101.8	1.3	0.05
6	1119.5	1119.4	1119.2	0.3	0.01
7	1099	1098.8	1098.6	0.4	0.02

Supplementary Table S2. Raw data for the field trials for the evaluation of sequential generation of aliquots of sweat. Only 3 of 8 individuals produced enough sweat to fill the initial sweatainer (Sweatainer #1) to require replacement (Sweatainer #2). Chloride measurements obtained via colorimetric analysis from an image captured immediately preceding the removal of the sweatainer at the indicated time during the 50 min exercise period. Reported chloride concentrations and standard deviations reflect color measurements for N = 3 positions within a given chamber.

Participant #	Sweatainer #	Total Volume Collection (µL)	Time (min)	Chamber #	Chloride Concentration (mM)
1	1			1	5.5 ±0.6
				2	9.1 ± 0.7
		50.8	35	3	10.1 ± 0.9
	2			1	11.5 ± 0.3
		33.8	50	2	14.7 ± 1.2
2	1			1	7.6 ± 0.3
				2	9.7 ± 0.4
		50.8	28	3	11.2 ± 0.9
	2			1	17.1 ± 0.7
		42.3	50	2	25.3 ± 0.8
3	1			1	15.8 ± 0.8
				2	18.6 ± 0.3
		50.8	35	3	24.6 ± 0.7
	2	31.5	50	1	27.6 ±0.1
4	1			1	10.0 ± 0.3
		41.4	50	2	16.1 ± 0.4
5	1			1	11.5 ± 0.6
		42.1	50	2	13.4 ± 0.6
6	1			1	19.5 ± 1.0
		37.8	50	2	21.6 ± 0.4
7	1			1	10.4 ± 0.5
		34.2	50	2	18.6 ± 1.0
8	1			1	8.7 ± 0.8
		33.8	50	2	9.9 ± 0.4
